# Morphological residual convolutional neural network (M-RCNN) for intelligent recognition of wear particles from artificial joints

**DOI:** 10.1007/s40544-021-0516-2

**Published:** 2021-08-18

**Authors:** Xiaobin Hu, Jian Song, Zhenhua Liao, Yuhong Liu, Jian Gao, Bjoern Menze, Weiqiang Liu

**Affiliations:** 1 Department of Computer Science, Technical University of Munich, Garching, 85748 Germany; 2 School of Biomedical Engineering, Sun Yat-sen University, Guangzhou, 510006 China; 3 Key Laboratory of Biomedical Materials and Implant Devices, Research Institute of Tsinghua University in Shenzhen, Shenzhen, 518057 China; 4 State Key Laboratory of Tribology, Tsinghua University, Beijing, 100084 China; 5 Mckelvey School of Engineering, Washington University in Saint Louis, St. Louis, MO 63130 USA

**Keywords:** wear particles classifier, morphological priors, data augmentation, deep residual network

## Abstract

Finding the correct category of wear particles is important to understand the tribological behavior. However, manual identification is tedious and time-consuming. We here propose an automatic morphological residual convolutional neural network (M-RCNN), exploiting the residual knowledge and morphological priors between various particle types. We also employ data augmentation to prevent performance deterioration caused by the extremely imbalanced problem of class distribution. Experimental results indicate that our morphological priors are distinguishable and beneficial to largely boosting overall performance. M-RCNN demonstrates a much higher accuracy (0.940) than the deep residual network (0.845) and support vector machine (0.821). This work provides an effective solution for automatically identifying wear particles and can be a powerful tool to further analyze the failure mechanisms of artificial joints.

**Electronic Supplementary Material:**

Supplementary Material is available in the online version of this article at 10.1007/s40544-021-0516-2.

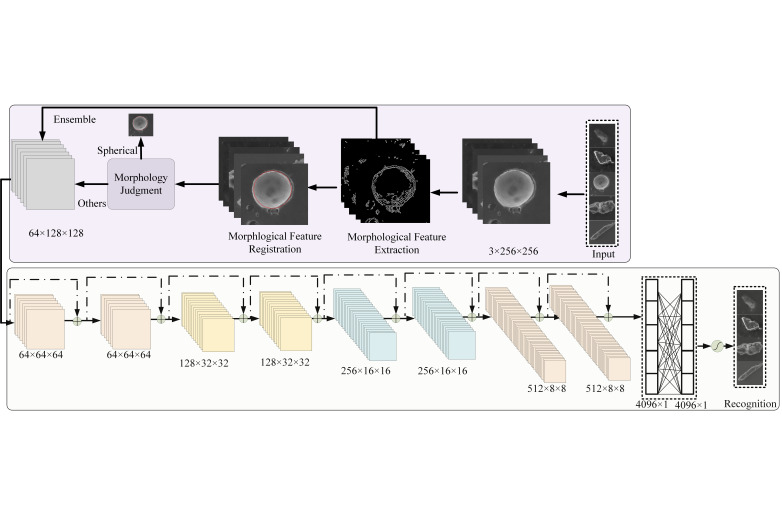

## 1 Introduction

The coronavirus disease (COVID-19) pandemic in 2020 has made people care more about health problems. Indeed, not only these epidemic diseases but also chronic diseases should be brought to the forefront. Osteoarthritis (OA), mainly caused by the degeneration of joints, is one of the chronic diseases that has affected the individual quality of life both physically and socially. To treat severe cases, artificial joints, e.g., artificial hip joint [1], artificial knee joint [2], and artificial disc [3], are needed to replicate the functionality of a normal and healthy joint [4]. Generally, the lifespan of artificial joints is designed to be 20–40 years [5, 6]. However, after long-term service in the human body, the articular surfaces will be worn and thus generate wear particles. It has been reported that the wear particles of artificial joint prosthesis may induce host response in the human body and cause a so-called "particle disease" [7]. Clinically, wear particles in the size of micrometers or less can lower bone density through osteolysis and further lead to aseptic loosening [8], making particle related disease one of the top leading causes for prosthesis failure after long-term implantation [9]. Therefore, it is crucial to carry out failure analysis through identifying wear particles whose shape and surface morphologies are closely related to their generation mechanisms and wear conditions.

However, analysis of these wear particles is a complicated task. Since the artificial joints are usually lubricated by tissue fluids (*in vivo*) or simulated body fluids (*in vitro*), extra isolation is always needed to remove the biomacromolecules impurities and purify the wear particles before characterization [10]. With several digestion and isolation protocols, scientists have evaluated the wear particles produced by different artificial joints recently. McMullin et al. [11] analyzed 750 wear particle images from the periprosthetic failed hip, knee, and shoulder arthroplasties, and based on particle morphologies and according to commonly used terminology, classified them into three groups, i.e., fibers, flakes, or granules. Liu et al. [12] compared the wear particles generated from the implanted artificial joint (*in vivo*) and joint simulator (*in vitro*). They indicated that the typical morphologies of wear particles were spherical, block, tear, sheet, rod, and strip. In our previous study [13], we also investigated the wear particles of artificial disc and summarized their representative morphologies as flake-like wear particles, spherical wear particles, aggregated wear particles, rod-like wear particles, and zonal wear particles. Indeed, each type of wear particle contains its specific information about wear processes and wear mechanisms. For instance, flake-like particles are contributed by abrasive wear and fatigue wear, spherical particles can be attributed to adhesion wear, aggregated particles are the products of several mechanisms, and rod-like and zonal particles are dominated by peeling and fragmentation after fatigue wear [13]. Therefore, finding the correct category of the wear particle not only helps understand the morphology of the particle itself, but also gives the information about wear status and wear mechanisms of the artificial joints [14].

Although the investigations mentioned above are good examples of how recent studies have evaluated the wear particles generated by artificial joints, most of them are recognized manually. It has been proposed that up to $2 \times 10^8$ wear particles are produced per year by the implanted artificial joint in patients [15]. Thus, we may be still far away from appreciating the full range of wear particles for which more useful wear information can be provided. Indeed, with the development of artificial intelligence, some neural networks have been employed to establish wear particle classifiers [16–19]. For instance, a multi-level belief rule base system [20] and a linear support vector machine [21] were employed to optimize the wear information and classify wear particles. Wang et al. [17] proposed a two-level classification procedure: the first-level to determine the three classes by a back-propagation network and the second-level to further identity fatigue particles and severe sliding particles by a six-layer convolutional neural network. Peng et al. [22] developed a hybrid convolution neural network that mainly consisted of transfer learning and support vector machine techniques to identify four types of wear debris. However, most of them are not designed for the wear particles of artificial joints, and their efficiency and accuracy still need to be improved.

In addition to the differences highlighted above, another specific point of wear particles from artificial joints that we should note is the imbalanced distribution of particle types. Compared to other industry cases, the applied load and resulting-in wear rates are much smaller, i.e., wear particle harvest is more difficult and a smaller amount can be collected. Therefore, the imbalanced distribution of particle types may be more prominent. It is suggested that the subsphaeroidal wear debris from the artificial hip joints took up the majority of the debris but shared a smaller percentage of the total volume, indicating the type distribution was imbalanced [12]. Eckold et al. [23] suggested that the majority of the morphological occurrences of wear particles from artificial discs was fibril. Regarding the artificial knee and hip joints, it was concluded that the particle morphology can be strongly influenced by the lack of experimental precision with different quantitative approaches and the *in vivo* wear particle distribution was not homogeneous owing to the clumping and clearing of particles through drainage [10]. When being recognized by a neural network, such imbalanced distribution poses a challenge for predictive modeling, as most of the machine learning algorithms used for classification were designed around the assumption of an equal number of examples for each class, meaning normal models may demonstrate poor predictive performance, especially for the minority ones [24]. Therefore, a specific recognition model is required to address the imbalanced sample size issue, which affects the accuracy of particle classification using a machine learning approach.

Based on the wear particles we obtained in our previous work [13] and motivated by the considerations above, in this paper, we propose a morphological residual convolutional neural network (M-RCNN) as an automatic wear particle classification model to tackle the extremely class imbalance problem. Specifically, the morphological priors are extracted using a Canny detector, which then uses them to pick out spherical wear particels by matching them to Houghcircle. To further alleviate the data imbalance problem, the remaining four highly geometric similarity classes—the flake, aggregated, rod-like, and zonal wear particles—are used to synthesize more training images by the data augmentation technique. Finally, all training images, including the synthesized images and their corresponding morphological priors, are imported into the residual convolutional neural network to extract the key features and then adaptively train a classification model.

The rest of the paper is organized as follows. The details of data preprocessing and data augmentation are illustrated in Section 2 to alleviate the class imbalance problem. The working principle of wear particle classification using the morphological residual convolutional neural network (M-RCNN) is presented in the same section. Afterwards, Section 3 describes two experiments to examine the reliability and accuracy of the proposed method as well as discussion on the presented results. Finally, the conclusions are drawn in Section 4.

## 2 Recognition network construction

### 2.1 Datasets and implementation details

Wear particles were generated and isolated from our previous *in vitro* wear simulations. Those wear particles were isolated and then imaged using scanning electron microscopy (SEM, FEI Quanta 200FEG, Eindhoven, The Netherlands) as presented in Ref. [13]. Prior to the SEM observation, a thin platinum (Pt) layer was coated onto the samples to improve the image quality. The schematic of the particle imaging system is demonstrated in Fig. 1. In this study, we collected ~900 images of wear particles, 80% of the samples were randomly selected as the training dataset while the rest (20%) were used for testing. As mentioned above, the distribution of the wear particles we obtained is inhomogenous. More specifically, the ratio of the maximum class samples to the minimum class samples is 15.8 (flake 553: spherical 35: aggregated 170: rod-like 69: zonal 106). For implementation details, an adaptive moment estimation (ADAM) optimizer is used with a batch size of 64 for training [25]. The initial learning rate is set to be 0.0005, which is divided by 2 every 100 epochs. The whole training process takes 1,000 epochs with an NVIDIA Titan X GPU.

### 2.2 Data preprocessing and augmentation

Since data preprocessing plays an important role in our overall framework, we here aim to employ a

**Fig. 1** Diagram of the particle imaging system.

simple but effective image preprocessing on both the training and held-out testing sets. The main motivation of data augmentation is to help our network to grasp more typical features and then build up a good mapping function by creating and exploring more variants of images (e.g., geometric and color transformations). The data augmentation does not ruin the input pixel distribution which is vital information for the network to learn but only boost the data diversity especially for the minimum class. The process of data augmentation is guided by the following three steps. In order to ensure a uniform image size of all data imported into the M-RCNN in the training and test stages, we crop and downsample each subject to a uniform 256×256 image size with three channels. Secondly, we implement the Gaussian normalization on the pixels' intensity whose range changes from [0, 255] to [–1, 1]. This step aims to normalize the pixel intensity and further reduce variation among the pixels to avoid the performance deterioration of the model stemming from the non-uniform data range. Finally, we endow the classification model with the desired invariance and robustness for class imbalance problems by performing data augmentation to synthesize more input images that better represent input distribution.

Indeed, data augmentation is an effective way to equip the model with the ability to resist the training data imbalance and scarcity. More information can be extracted from the original dataset through data augmentation. This inflates the training dataset size by data warping which transforms existing images while their label is preserved. The warp augmentation encompasses geometric and color transformations, random erasing, adversarial training, and neural style transfer. In the case of wear particles among different classes, due to the variations of camera orientations, resolution, and noise, we primarily perform the rotation, flip, noise, affine, and blur transformations (Fig. 2). The rotation degree is in a range of [–90°, 90°] and represents the variation of camera orientations. A 9×9 kernel is used to blur images to highlight their shape information and weaken texture information. Meanwhile, we add random noise to images to make the model more stable and robust [26].

For data preprocessing, the input images are center-cropped as RGB channels. After the data preprocessing, we can obtain a dataset that is three times larger than the original one and the imbalanced distribution problem is alleviated to some extent.

### 2.3 Morphological residual convolutional neural network (M-RCNN)

The convolutional neural network (CNN) has proven to be an effective computational model for automatically extracting image features [27–29]. A morphological-based convolutional neural network is constructed to grasp more morphological priors, which then guides the network to categorize the input wear particles images. Consequently, two parts are involved in this model: a morphological-priors generation block and a convolutional neural network classifier. The reasons for worse classification performance usually come from two aspects: (1) lack of reliable priors to guide

**Fig. 2** An example of data augmentation. From left to right: the original axial slice, slice after rotation, slice after shear mapping, and slice after scaling. After data augmentation, we can obtain a dataset three times larger than the original one while the class imbalance problem is alleviated to some extent.

the classifier to make a reasonable judgment; (2) the knowledge interference caused by inhomogeneous distribution among classes. Thus, the main motivation of morphological-priors is to embed the network or classifier with more faithfully shape-based knowledge that is greatly different among various swear particles.

For the morphological-priors generation block, we apply a Sobel Kernel framework to extract the shape-priors. Specifically, we utilize a Gaussian filter to blur and smooth the image to suppress random and sharp noise that can affect the mainly shape-based edge extraction of wear particles. Then the intensity gradients of images are traced to delineate the complete shape of wear particles. The smoothed image is filtered with a Sobel Kernel in both horizontal and vertical directions to get the first derivate in the horizontal direction ($G_x$) and vertical direction ($G_y$). Then, we find the edge gradient and direction for each pixel as follows:

$$\text{Edge\_Gradient}(G) = \sqrt{G_x^2 + G_y^2}$$
$$\theta = \tan^{-1}\left(\frac{G_y}{G_x}\right) \tag{1}$$

Non-maximum suppression is executed to avoid the spurious response and then double threshold is to determine potential edges. The final shape is determined by suppressing all the edges which are weak and not connected to strong edges.

In a next step, we employ a Canny detector to extract useful structural information (e.g., shape-based boundary and morphological-based texture) and highlight the shape information. Specifically, Houghcircle, controlled by the parameters including minimum and maximum circle radius and minimum distance between the centers of the detected houghcircles, is registered and matched into the morphological space. After the houghcircle registration, the morphological judgment block picks out the highest geometric discrepancy class (spherical wear particles in our case).

In the following CNN-based classifier, wear particle images and their corresponding morphological priors are imported into the classifier. The classification model is mainly composed of three layers: convolutional layer, residual connection layer, and fully-connected layer. The convolutional layer is to characterize image features by local receptive fields and shared weights. The motivation for residual connection over layers is to avoid the problem of vanishing gradients, by reusing activations from a previous layer until the adjacent layer learns its weights. After the full connection operation, all the local features are integrated for object recognition. By doing so, these three layers can provide shift, scale, and distortion invariance of the extracted features.

As shown in Fig. 3, wear particle images are cropped and downsampling in a uniform size of 256 × 256 × 3. The effective combination of convolutional

**Fig. 3** The proposed intelligent recognition architecture of wear particles. This model consists of two branches: the top block is a feature extractor to grasp the morphological priors and the bottom block is dedicated to wear particles recognition by employing the residual learning.

layer, batch normalization, and rectified linear unit is repeatedly used in the model. The information transformation process of the convolutional layer is executed by a filtering operation. The convolutional transformation is described as follows:

$$O=\left[\frac{H-H_{\mathrm{k}}+2p}{S}+1, \frac{W-W_{\mathrm{k}}+2p}{S}+1, n\right] \tag{2}$$

where $H$ and $H_{\mathrm{k}}$ are the height size of input features and kernel, respectively. Accordingly, $W$ and $W_{\mathrm{k}}$ are the width size of input features and kernel. $p$ and $S$ denote the padding and stride size, and $n$ stands for the RGB channels.

The distribution of each layer's inputs can change during training, leading to the internal covariate shift [30]. Thus, we perform the batch normalization for each training min-batch. Here, batch normalization acts as a regularizer, which allows us to use much higher learning rate and relax the restriction of the initialization. For a layer with d-dimensional input $\boldsymbol{x}=(x^1 \ldots x^d)$, the batch normalizing transform is illustrated as follows:

$$\begin{aligned}
&y_i=\mathrm{BN}_{\gamma,\beta}(x_i)\\
&\mu_B \leftarrow \frac{1}{m}\sum_{i=1}^{m} x_i\\
&\sigma_B^2 \leftarrow \frac{1}{m}\sum_{i=1}^{m}(x_i-\mu_B)^2\\
&\hat{x}_i \leftarrow \frac{x_i-\mu_B}{\sqrt{\sigma_B^2+\varepsilon}}\\
&y_i \leftarrow \gamma\hat{x}_i+\beta \equiv \mathrm{BN}_{\gamma,\beta}(x_i)
\end{aligned} \tag{3}$$

where $\mu_B, \sigma_B$ denote the mean and variance of d-dimensional input $\boldsymbol{x}$. $\hat{\boldsymbol{x}}$ is the normalized output. For each activation $\hat{x}^i$, a pair of parameters $\gamma$ and $\beta$ are the scale and shift processing of the normalized $\hat{\boldsymbol{x}}$. These parameters are learned along with the original model parameters. By setting $\gamma=\sqrt{\mathrm{Var}[\boldsymbol{x}]}$ and $\beta=E[\boldsymbol{x}]$, it could recover the original activations.

The sigmoid and hyperbolic tangent activation functions cannot be used in networks with many layers due to the vanishing gradient problem and saturate phenomena, while the rectified linear activation function can overcome the limitations, allowing models to learn faster and perform better [31]. Therefore, in our model, the rectified linear unit activation function is inserted behind convolutional layers and batch normalization layers as follows [32]:

$$R(\boldsymbol{x})=\max(0, \boldsymbol{w}^{\mathrm{T}}\boldsymbol{x}+\boldsymbol{b}) \tag{4}$$

where $R$ is the function output, $\boldsymbol{x}$ is the input imported into the convolutional layer, and $\boldsymbol{w}$ and $\boldsymbol{b}$ denote the weight matrix and bias of convolution layers, respectively.

In addition, to solve the problem of vanishing gradients, we adopt residual learning to the stacked layers (Fig. 3). A neural network without residual parts explores more of the feature space. This makes it more vulnerable to perturbations that cause it to leave the manifold, and necessitates extra training data to recover. After introducing the skipping connection, on the contrary, it simplifies the network effectively and speeds the learning by reducing the impact of vanishing gradients and using the shallower layers. The network then gradually restores the skipped layers as it learns the feature space. Towards the end of the training, when all layers are expanded, it stays closer to the manifold and thus learns faster. Formally, we consider a building block defined as:

$$\boldsymbol{y}=\boldsymbol{F}(\boldsymbol{x},\{W_i\})+\boldsymbol{x} \tag{5}$$

where $\boldsymbol{x}$ and $\boldsymbol{y}$ are the input and output vectors of the layers considered. The function $\boldsymbol{F}(\boldsymbol{x},\{W_i\})$ denotes the residual mapping to be learned. Here, we assume that the function $\boldsymbol{F}$ has two layers, then $\boldsymbol{F}=W_2\sigma(W_1\boldsymbol{x})$ in which $\sigma$ means the activation function and biases are omitted for simplifying notations. The operation $\boldsymbol{F}+\boldsymbol{x}$ is performed by a shortcut connection and element-wise addition. The shortcut connection is attractive in practice because it does not introduce extra parameter and computation complexity.

The dimensions of $\boldsymbol{x}$ and $\boldsymbol{F}$ must be equal in Eq. (5). If this is not the case (e.g., when changing the input/output channels), we can perform a linear projection $W_s$ by the shortcut connections to match the dimensions:

$$\boldsymbol{y}=\boldsymbol{F}(\boldsymbol{x},\{W_i\})+W_s\boldsymbol{x} \tag{6}$$

In a next step, the multi-class identification of wear particles is implemented by softmax function, a last activation function to normalize the output of a network to a probability distribution over predicted output classes [33]:

$$P(Y=i\mid \boldsymbol{R},\boldsymbol{w},\boldsymbol{b})=\operatorname{soft}\max(\boldsymbol{wR}+\boldsymbol{b})=\frac{e^{w_i R+b_i}}{\sum_j e^{w_i R+b_i}} \quad (7)$$

where $\boldsymbol{R}$ is a given input feature vector, $\boldsymbol{w}$ and $\boldsymbol{b}$ are a weight and bias vector. $i$ is the input belongs to the $i$-th category of particles and $j$ is the category index. The probability summation of different categories $\sum P$ should be 1. The maximum probability value of all categories is regarded as the corresponding particle type.

To measure the distance between the prediction of wear particle and its true labeled value, we construct a multi-categorical cross-entropy loss as follows [32]:

$$\text{loss}=-\sum_{i=1}^{j} y_i \log f_i(\boldsymbol{x}) \quad (8)$$

where $\boldsymbol{x}$ is the input of wear particles and $j$ is the number of classes. $y_i$ is the true label of $i$-th class and $f_i(\boldsymbol{x})$ is the prediction output of the classification model. Based on this, model weights and offsets are iteratively updated and learned until the objective function reaches a satisfactory value.

Moreover, we combine the predictions of the original model and morphological model to improve the generalizability and robustness. The original model means that the inputs are four original class images of wear particles where the morphological priors are only used to pick out the spherical particles. Different from the original model, the input of the morphological model is only morphological images (as shown in morphological feature extraction in Fig. 3) without original images. For orginal model and morphological model, they both first take out the spherical particles from five classes and make a classification on remaining four classes wear particles. The particles identification can be summarized as:

$$\begin{aligned}\boldsymbol{P}_1^{c=4}&=\text{Net}(\boldsymbol{o})\\ \boldsymbol{P}_2^{c=4}&=\text{Net}(\boldsymbol{m})\\ \boldsymbol{P}&=W_1\boldsymbol{P}_1^{c=4}+(1-W_1)\boldsymbol{P}_2^{c=4}\end{aligned} \quad (9)$$

where $c$ is the four classes of wear particles, $P_1$ is a 4 × 4 matrix obtained by the original model, and $\boldsymbol{P}_2$ is a 4 × 4 matrix obtained by the morphological model. $\boldsymbol{o}$ and $\boldsymbol{m}$ are the input of original and morphological wear particles, respectively. Each element of $\boldsymbol{P}$ means the credible score belongs to the specific class. $W_1$ and 1–$W_1$ are the weight of models $\boldsymbol{P}_1$ and $\boldsymbol{P}_2$, and we set the $W_1$ as 0.9. Finally, the final prediction of classes is a weighted summation with two models.

## 3 Experiments and discussion

### 3.1 Performance evaluation of particle recognition network

We analyze our morphological residual convolutional neural network (M-RCNN) on the 181 unseen test data to show our superiority compared with the conventional networks, i.e., support vector machine (SVM), deep residual network (ResNet), and deep residual network with data augmentation (ResNet+Aug) models. To evaluate the recognition accuracy, the precision, recall and F1-score values are calculated. Their expressions are described as follows:

$$\begin{aligned}\text{Accuracy}&=\frac{\text{TP}+\text{TN}}{\text{TP}+\text{FP}+\text{FN}+\text{TN}}\\ \text{Precision}&=\frac{\text{TP}}{\text{TP}+\text{FP}}\\ \text{Recall}&=\frac{\text{TP}}{\text{FN}+\text{TP}}\\ \text{F1}&=\frac{2\times\text{precision}\times\text{recall}}{\text{precision}+\text{recall}}\end{aligned} \quad (10)$$

where TP, TN, FN and FP are the number of true positive, true negative, false negative, and false positive, respectively.

For comparative analysis, the recognition accuracy, precision, recall, and F1-score criteria of different configurations are listed in Table 1 and Table S1 in the Electronic Supplementary Material (ESM). It indicates the SVM and ResNet models are not able to identify the minimum class (spherical wear particles) which suffered by very limited data, only 4% of whole database. Besides, after embedding the data augmentation, ResNet+Aug model improves its precision slightly from 0.612 to 0.649, but still fails to distinguish the high-geometric similarity classes among

**Table 1** The precision and accuracy values of wear particle recognition using different methods: support vector machine (SVM), deep residual network (ResNet), deep residual network with data augmentation (ResNet+Aug), and morphological residual convolutional neural network (M-RCNN). A, B, C, D, and E donate the flake-like, spherical, aggregated, rod-like, and zonal particles, respectively.

| | Wear particle | SVM | ResNet | ResNet+Aug | M-RCNN |
|---|---|---|---|---|---|
| Precision | A | 0.584 | 0.632 | 0.656 | 0.855 |
| | B | NAN | NAN | NAN | 0.714 |
| | C | 0.250 | 0.545 | 0.667 | 0.821 |
| | D | 0 | 0.500 | 0.615 | 0.857 |
| | E | 0 | 0 | 0.333 | 0.933 |
| | Overall | 0.553 | 0.612 | 0.649 | 0.851 |
| Accuracy | A | 0.559 | 0.617 | 0.670 | 0.872 |
| | B | 0.963 | 0.963 | 0.963 | 0.979 |
| | C | 0.787 | 0.835 | 0.840 | 0.915 |
| | D | 0.915 | 0.926 | 0.941 | 0.979 |
| | E | 0.883 | 0.883 | 0.883 | 0.957 |
| | Overall | 0.821 | 0.845 | 0.860 | 0.940 |

the zonal, aggregated and flake-like wear particles. Regarding the M-RCNN model we developed in this study, it achieves the best performance in all criteria including the precision (0.851), recall (0.851), F1-measure (0.851), and accuracy (0.940) compared with other conventional methods. This method not only discriminates the high-geometric similarity classes but also effectively picks out the minimum class (spherical wear particles in this study) using the morphological priors for the extremely class imbalance problem. To evaluate the efficiency, we calculate the computation test time of each image on a server with an Intel Xeon W-2123 CPU and an NVIDIA TITAN X GPU. The obtained results indicated that the computation test time of M-RCNN is 4.7 ms, which is similar to the reported ones [22], indicating the algorithms we proposed achieve state-of-the-art accuracy and still keep a comparable test time.

To evaluate the classifiers' quality, the multi-class receiver operating characteristic curves (ROC) of different models are shown in Fig. 4, which is created by plotting the true positive rate (TP) against the false positive rate (FP) at various threshold settings. For the micro-average method, it computes the metrics based on the global confusion matrix to get the true positives, false positives, false negatives, and true negatives of the system, by assigning equal weight on each data (not each class). The macro-average calculates the metrics independently based on the local confusion matrix of each class and then takes the average, assuming that the weight or contribution of each class is equal. The closer ROC value to 1, the better a classifier is [34]. As can be seen in Fig. 4, although data augmentation has been applied to the ResNet model, its macro-average ROC area is only 0.68, which is just 71.6% of our M-RCNN algorithm. Indeed, the M-RCNN demonstrates the best performance from the ROC point of view. In addition, as presented in Fig. 5, the features of wear particles extracted both by ResNet and ResNet+Aug models are mixed while the M-RCNN model we developed here can clearly separate the four resting classes in a large distance, indicating an excellent performance of our morphological model on features extraction.

### 3.2 Performance evaluation of morphological priors

In this section, we aim to demonstrate the effectiveness of the morphological priors. Here we compare the proposed network with and without using the morphological priors in terms of precision and accuracy values (see Eq. (10) for details) which are listed in Table 2 and Table S2 in the ESM. Although the precision and accuracy of M-RCNN model without morphological priors are relatively high compared to the conventional models, applying the morphological priors can further boost both the precision (from

**Fig. 4** Receiver operating characteristic curves (ROC) with different configurations: (a) M-RCNN , (b) ensembled model, (c) M-RCNN without morphological knowledge, (d) ResNet without data augmentation, and (e) ResNet with data augmentation.

**Table 2** The ablation study of wear particle recognition using our different schemes: with/without morphological priors and with/without ensemble mechanism: M-RCNN without morphological priors, M-RCNN and ensembled models. A, B, C, D, and E donate the flake-like, spherical, aggregated, rod-like, and zonal particles, respectively.

| | Wear particle | M-RCNN without morphological priors | M-RCNN | Ensembled |
|---|---|---|---|---|
| Precision | A | 0.811 | 0.855 | 0.856 |
| | B | 0.571 | 0.714 | 0.714 |
| | C | 0.806 | 0.821 | 0.852 |
| | D | 0.818 | 0.857 | 0.857 |
| | E | 0.750 | 0.933 | 0.933 |
| | Overall | 0.798 | 0.851 | 0.856 |
| Accuracy | A | 0.824 | 0.872 | 0.878 |
| | B | 0.968 | 0.979 | 0.979 |
| | C | 0.920 | 0.915 | 0.920 |
| | D | 0.963 | 0.979 | 0.979 |
| | E | 0.920 | 0.957 | 0.957 |
| | Overall | 0.919 | 0.940 | 0.943 |

0.798 to 0.851) and accuracy (from 0.919 to 0.940) of M-RCNN model. We can also observe from Fig. 4, the macro-average ROC area of M-RCNN is improved by nearly 10% after adding the morphological priors. Regarding the feature extraction capability, as presented in Figs. 5(a) and 5(c), the model exploiting the morphological priors performs a stronger ability in extracting and identifying the wear particle features.

Since aggregated particles have similar geometric characters as flake-like and zonal particles, it is difficult to split them from the other two categories. Thus, we also ensemble two models in this study: the one is based on the morphologic priors input and the other is on the original wear particle samples. The obtained

**Fig. 5** Dimensionality reduction results of the features extracted by different methods using the t-SNE algorithm: (a) M-RCNN, (b) ensembled model, (c) M-RCNN without morphological knowledge, (d) ResNet without data augmentation, and (e) ResNet with data augmentation. “t-SNE” donates “t-Distributed Stochastic Neighbor Embedding”, which is a non-linear dimensionality reduction algorithm used to analyze or visualize high-dimensional data by mapping multi-dimensional data to two or more dimensions that is better for human observation.

results indicate that incorporating morphologic models can not only enable the algorithm to get a better recognition (3% higher) on the similar wear particles classes, but also make the accuracies of other criteria do not deteriorate.

### 3.3 Outlook

M-RCNN developed in this study is tailored based on real scenarios of wear particles from artificial joints, breaking the imbalance barrier that each type of wear particle appears in largely different numbers. Since our M-RCNN well exploits the morphological priors, it is better to apply it to other scenarios in which each type of class presents different distinct-geometry morphology. However, owing to the limited numbers of wear particles we collected in our previous studies, this model has not been well trained to some extend. Future investigations could continue to analyze the generalization ability of M-RCNN by collecting much more wear particles or testing in other similar wear particles data. Another direction of interest lies in the GANs-based data augmentation, which is also a potential way to enhance the diversity of input data beyond the operation of rotation and flip transformations.

## 4 Conclusions

Recognition and understanding the morphology of wear particles generated from artificial joints are of importance to study their wear mechanisms and further improve the wear performance. One of the biggest challenges of the task is the imbalance distribution of each group. In this paper, we proposed a morphological residual convolutional neural network (M-RCNN) incorporating the morphological priors to solve the recognition problem under inhomogeneous distribution conditions. The morphological priors are extracted by the Canny detector to highlight useful structural and shape information, and then matched by the specific shape (spherical wear particles in this study) to pick out the geometry-different class. To further alleviate the data imbalance problem, the data augmentation technique is utilized to synthesize more training samples of the high geometric similarity classes. The M-RCNN is tested using a large number of wear particles images to show its better performance as compared with several available classification models which are suffered from class imbalance problems. Therefore, M-RCNN is a promising tool for the automatic artificial joint wear particle classification no matter how the distribution is and has the potential for applications in further tribological analysis of artificial joints.

## Acknowledgements

This work is financially supported by the National Natural Science Foundation of China (No. 51875303). Support through the start-up foundation from Sun Yat-sen University is also gratefully acknowledged. Xiaobin Hu acknowledges the funding from the China Scholarship Council (CSC).

**Electronic Supplementary Material:** Supplementary Material is available in the online version of this article at https://doi.org/10.1007/s40544-021-0516-2.

**Open Access** This article is licensed under a Creative Commons Attribution 4.0 International License, which permits use, sharing, adaptation, distribution and reproduction in any medium or format, as long as you give appropriate credit to the original author(s) and the source, provide a link to the Creative Commons licence, and indicate if changes were made.

The images or other third party material in this article are included in the article's Creative Commons licence, unless indicated otherwise in a credit line to the material. If material is not included in the article's Creative Commons licence and your intended use is not permitted by statutory regulation or exceeds the permitted use, you will need to obtain permission directly from the copyright holder.

To view a copy of this licence, visit http://creativecommons.org/licenses/by/4.0/.

## Electronic Supplementary Material

Morphological residual convolutional neural network (M-RCNN) for intelligent recognition of wear particles from artificial joints
