# Supplementary material for: Morphological residual convolutional neural network (M-RCNN) for intelligent recognition of wear particles from artificial joints
Source: Friction. 2021 Aug 18;10(4):560–72. doi: 10.1007/s40544-021-0516-2 (PMC8372229; doi:10.1007/s40544-021-0516-2)
Supplement: Supplementary file 1 — Morphological residual convolutional neural network (M-RCNN) for intelligent recognition of wear particles from artificial joints [file 40544_2021_516_MOESM1_ESM.pdf]

Electronic Supplementary Material

Morphological residual convolutional neural network (M-RCNN) for intelligent recognition of wear particles from artificial joints

Xiaobin HU[1], Jian SONG[2,*], Zhenhua LIAO[3], Yuhong LIU[4,*], Jian GAO[5], Bjoern MENZE[1], Weiqiang LIU[3,4]

[1] *Department of Computer Science, Technical University of Munich, Garching 85748, Germany*

[2] *School of Biomedical Engineering, Sun Yat-sen University, Guangzhou 510006, China*

[3] *Key Laboratory of Biomedical Materials and Implant Devices, Research Institute of Tsinghua University in Shenzhen, Shenzhen 518057, China*

[4] *State Key Laboratory of Tribology, Tsinghua University, Beijing 100084, China*

[5] *Mckelvey School of Engineering, Washington University in Saint Louis, St. Louis, MO 63130, USA*

Supporting information to https://doi.org/10.1007/s40544-021-0516-2

Table S1 The true positive (TP), true negative (TN), false negative (FN), false positive (FP), recall, and F1-score values of different models: support vector machine (SVM), deep residual network (ResNet), deep residual network with data augmentation (ResNet+Aug), and morphological residual convolutional neural network (M-RCNN). A, B, C, D, and E donate the flake-like, spherical, aggregated, rod-like, and zonal particles, respectively.

Model	Wear particle	TP/FP	TN/FN	Recall	F1-measure
SVM	A	101/72	4/11	0.902	0.709
	B	0/0	181/7	0	0
	C	3/9	145/31	0.088	0.130
	D	0/2	172/14	0	0
	E	0/1	166/21	0	0
	Overall	104/84	668/84	0.553	0.553
ResNet	A	96/56	20/16	0.857	0.727
	B	0/0	181/7	0	0
	C	18/15	139/16	0.529	0.537
	D	1/1	173/13	0.071	0.125
	E	0/1	166/21	0	0
	Overall	115/73	679/73	0.612	0.612
ResNet+Aug	A	105/55	21/7	0.938	0.772
	B	0/0	181/7	0	0
	C	8/4	150/26	0.235	0.348
	D	8/5	169/6	0.571	0.593
	E	1/2	165/20	0.048	0.083
	Overall	122/66	686/66	0.649	0.649
M-RCNN	A	106/18	58/6	0.946	0.898
	B	5/2	179/2	0.714	0.714
	C	23/5	149/11	0.676	0.742
	D	12/2	172/2	0.857	0.857
	E	14/1	166/7	0.667	0.778
	Overall	160/28	724/28	0.851	0.851

* Corresponding authors: Jian SONG, E-mail: songj67@mail.sysu.edu.cn; Yuhong LIU, E-mail: liuyuhong@tsinghua.edu.cn

Table S2 The ablation study of wear particle recognition using different schemes: with/without morphological priors and with/without ensemble mechanism. The true positive (TP), true negative (TN), false negative (FN), false positive (FP), recall, and F1-score values of different methods: M-RCNN without morphological priors, M-RCNN, and ensembled models. A, B, C, D and E donate the flake-like, spherical, aggregated, rod-like, and zonal particles, respectively.

Model	Wear particle	TP/FP	TN/FN	Recall	F1-measure
M-RCNN without morphological priors	A	103/24	52/9	0.920	0.862
	B	4/3	178/3	0.571	0.571
	C	25/6	148/9	0.735	0.769
	D	9/2	172/5	0.643	0.720
	E	9/3	164/12	0.429	0.545
	Overall	150/38	714/38	0.798	0.798
M-RCNN	A	106/18	58/6	0.946	0.898
	B	5/2	179/2	0.714	0.714
	C	23/5	149/11	0.676	0.742
	D	12/2	172/2	0.857	0.857
	E	14/1	166/7	0.667	0.778
	Overall	160/28	724/28	0.851	0.851
Ensembled model	A	107/18	58/5	0.955	0.903
	B	5/2	179/2	0.714	0.714
	C	23/4	150/11	0.676	0.754
	D	12/2	172/2	0.857	0.857
	E	14/1	166/7	0.667	0.778
	Overall	161/27	725/27	0.856	0.856
